# A New Extended Pareto Distribution: Statistical Properties, Estimation, and Applications

**DOI:** 10.1155/tswj/6572559

**Published:** 2026-07-29

**Authors:** Merga Abdissa Aga

**Affiliations:** ^1^ Department of Statistics, Salale University, Fiche, Oromia, Ethiopia, slu.edu.et

**Keywords:** acceptance–rejection algorithm, extended Pareto (EP), maximum likelihood estimation (MLE), modified Fréchet generator, Pareto distribution

## Abstract

The Pareto distribution is a fundamental model for heavy‐tailed data, but its rigid structure often fails to capture the diverse tail behaviors and hazard rate patterns observed in real‐world applications. To address this limitation, we propose a new extended Pareto (EP) distribution, obtained by applying the modified Fréchet generator to the classical Pareto baseline. This novel construction introduces an additional shape parameter that enhances tail flexibility and accommodates a wide range of hazard rate shapes, including decreasing, increasing, bathtub, and upside‐down bathtub forms. We derive several key distributional properties, including the probability density and cumulative distribution functions, quantile function, and moments. Parameter estimation is carried out using the method of maximum likelihood, and the asymptotic properties of the estimators are established. Monte Carlo simulations confirm the consistency and efficiency of the estimators. The practical utility of the EP model is demonstrated through applications to diverse real datasets, where it consistently outperforms classical Pareto and other Pareto‐type competitors based on likelihood‐based criteria and goodness‐of‐fit tests. The proposed distribution provides a powerful new tool for modeling heavy‐tailed phenomena in economics, finance, reliability, hydrology, and environmental sciences.

## 1. Introduction

The Pareto distribution is a cornerstone of heavy‐tailed modeling, originally introduced by Vilfredo Pareto to describe the distribution of wealth and income [1]. Because of its mathematical tractability and ability to capture power‐law behavior, it has become a standard tool in economics, finance, insurance and risk analysis, reliability engineering, and environmental science [2–6]. Its defining feature, a scale‐invariant tail with polynomial decay, makes it particularly suitable for data exhibiting extreme events or rare but high‐impact outcomes.

Nevertheless, the classical Pareto model can be too restrictive for many empirical datasets. Observed phenomena often display a variety of tail behaviors, skewness levels, and hazard‐rate shapes that cannot be adequately captured by the standard two‐parameter Pareto distribution [7]. To overcome these limitations, numerous generalizations and extensions have been proposed. Examples include the Lomax or Pareto Type II [8], beta‐Pareto [9], Kumaraswamy–Pareto (KP) [10], Marshall–Olkin Pareto [11], and transmuted Pareto families [12]. These distributions introduce additional shape parameters and consequently offer greater flexibility in capturing real‐world patterns.

In addition, recent studies have focused on improving tail estimation and inference for Pareto‐type distributions, particularly under bias and censoring. Robust and bias‐reduced estimation procedures have been proposed for extreme value index estimation [13–15], whereas penalized likelihood approaches have been developed for censored heavy‐tailed data [16]. Furthermore, flexible extensions of the generalized Pareto distribution (GPD) have been introduced to enhance tail modeling, including the extended generalized Pareto (GP) model [17] and its regression‐based extension [18]. More recent contributions also propose novel estimation strategies for Pareto‐type tails based on geometric records [19].

A powerful approach to constructing new distributions is through generator functions, which embed a baseline model into a broader functional framework [20]. The beta‐ [9], Kumaraswamy‐ [10], and transmuted‐generated [12] families are well‐known examples that have enriched classical models by controlling tail behavior and hazard‐rate patterns. In the context of lifetime modeling, related developments such as the weighted power Lindley distribution have also demonstrated the importance of flexible distributional forms for capturing diverse hazard‐rate behaviors and data heterogeneity [21]. Among more recent developments, the modified Fréchet generator (MFG) has attracted attention for its ability to produce distributions with highly flexible tail behavior and diverse hazard structures [22–27].

However, despite the growing literature on generator‐based models, existing studies provide no integration of the MFG with a Pareto baseline. The MFG itself is a modification of the classical Fréchet‐based generator and introduces an additional mechanism for tail stretching and compression. This modification enables sharper control over lower and upper tail weights compared with earlier generator families. To the best of our knowledge, combining the MFG with the Pareto model has not been explored, leaving a conceptual and practical gap in the family of Pareto‐type distributions.

Although several Pareto‐type extensions and generator‐based families have been proposed, many existing models still exhibit limited and coupled control over tail heaviness and hazard behavior. In particular, achieving substantial tail flexibility or nonstandard hazard shapes often requires introducing multiple additional parameters, leading to reduced parsimony and numerical instability in estimation. Moreover, previously proposed generators applied to Pareto baselines rely on unbounded transformations, which may complicate inference for highly heavy‐tailed data. These limitations motivate the exploration of bounded generator mechanisms within the Pareto framework.

This unexplored combination forms the key gap in the literature and provides the main motivation for the present study. By introducing the Extended Pareto (EP) distribution, we show that the resulting model possesses substantially enhanced tail flexibility, a wide range of density and hazard‐rate shapes, and the ability to model datasets that classical and GP forms fail to describe adequately. The proposed EP distribution also admits decreasing and increasing hazard functions under different parameter values, capabilities that are not simultaneously available in most two‐parameter Pareto‐type extensions.

In addition to filling a theoretical gap, the EP model offers practical advantages: It provides improved fit for heavy‐tailed data, more flexible extreme‐value behavior, and interpretable shape parameters, making it suitable for applications in economics, reliability, hydrology, environmental science, and financial risk analysis.

The key contributions of this study are threefold:

1. Definition and properties: We formally define the EP distribution using the MFG and derive its fundamental characteristics, including the probability density function (PDF), cumulative distribution function (CDF), quantile function, moments, hazard‐rate function, and additional structural properties such as stochastic ordering and entropy measures.
2. Inference: We develop maximum likelihood estimation procedures, derive the corresponding log‐likelihood function, and discuss numerical considerations in solving the likelihood equations.
3. Simulation and empirical evaluation: Through an expanded Monte Carlo simulation including multiple sample sizes, alternative initial values, hyperparameter justification, and comparison of different parameter‐estimation methods, we assess estimator performance under various conditions. We further apply the EP model to multiple real datasets and compare its performance against several competing Pareto‐type distributions using histogram‐PDF overlays, quantile plots, hazard‐rate plots, and Kaplan–Meier survival curves.

The remainder of the paper is organized as follows. Section 2 introduces the MFG and formally derives the EP distribution. In addition, Section 2 presents the main statistical properties. Section 3 develops inference methods and also provides the Monte Carlo study. Section 4 presents the real‐data applications with comprehensive graphical analyses. Section 5 concludes with limitations and future research directions.

## 2. Definition and Basic Properties

### 2.1. Pareto Distribution

Let *X* be a nonnegative random variable. Consider the classical Pareto distribution with scale (minimum) parameter *k* > 0 and shape parameter *β* > 0, whose CDF and PDF are given, respectively, by [5]

(1) F0x;β=10−kxβ,β>,x≥k,

where *k* is the scale parameter representing the minimum (lower bound) of the support, and *β* is the shape parameter controlling the heaviness of the tail. The corresponding PDF is

(2) f0x;β,k=βkβx−β+1,x≥k.

The parameter *k* plays a crucial role in determining the starting point of the distribution. To facilitate estimation and simplify inference, [28] recommend normalizing the data by dividing each observation by a preselected lower bound, effectively setting *k* = 1. Under this normalization, the CDF and PDF reduce to the simpler forms

(3) F0x;β=101−x−β,β>,x≥,

(4) f0x;β=βx−β+1,x≥1.

This standardized representation provides a convenient benchmark for developing new generalized or extended families of Pareto‐type models, including the proposed EP distribution introduced in the next section.

### 2.2. MFG

Let *G*(*x*) be a baseline CDF supported on *x* ≥ 1. The classical Fréchet‐type generator transforms *G*(*x*) through exponential tail modulation. The MFG further generalizes this idea by introducing a power parameter *α* > 0, allowing additional control over tail stretching and compression.

The MFG is defined by the CDF [22].

Fx;α=1−exp−Gxα1−e−1, x≥10, α>,

with corresponding PDF

fx;α=αgxGxα−1exp−Gxa1−e−1,

where *g*(*x*) = *G* ^′^(*x*).

Compared with earlier generator families, the MFG provides sharper control over both lower and upper tail behavior through the exponential‐power structure, making it particularly suitable for heavy‐tailed data.

### 2.3. EP Distribution

To introduce additional flexibility in modeling heavy‐tailed data, we extend the classical Pareto model by employing the MFG to the baseline Pareto distribution.

Applying this generator to the Pareto baseline yields the EP distribution:

(5) Fx;β,α=1−exp−1−x−βα1−e−1,x≥10,β,α>.

This model introduces an extra parameter *α* that controls tail thickness and skewness, allowing the EP distribution to model a wider variety of heavy‐tailed phenomena than the classical Pareto.

The PDF is obtained by differentiating *F*(*x*; *β*, *α*) with respect to *x*:

fx;β,α=d dxFx;β,α=11−e−1exp−1−x−βαα1−x−βα−1βx−β+1.

Thus,

(6) fx;β,α=βα1−e−1x−β+11−x−βα−1exp−1−x−βα,x≥1.

**Proposition 1.**

The extended Pareto distribution *f*(*x*; *β*, *α*) is a legitimate pdf.

fx;β,α≥01 for all x≥,

∫1∞fx;β,αdx=1.

**Proof 1.**

Since *β* > 0, *α* > 0, and *x* ≥ 1, each factor in *f*(*x*; *β*, *α*) is nonnegative.

Let $u={\left(1-x^{-\beta }\right)}^{\alpha },$

du=βαx−β+11−x−βα−1dx,

when *x* = 1, *u* = 0; as *x*⟶∞, *u*⟶1.

Therefore,

∫1∞fx;β,αdx=11−e−1∫01e−udu=1−e−11−e−1=1.

Hence, *f*(*x*; *β*, *α*) satisfies the two defining properties of a valid PDF.

#### 2.3.1. Survival Function

The survival (reliability) function is

(7) Sx;β,α=exp−1−x−βα−e−11−e−1.

This function captures the heavy‐tailed decay, with *α* adjusting how quickly the survival probability decreases.

#### 2.3.2. Hazard Function

The hazard rate, describing the instantaneous failure risk, is given by

(8) hx;β,α=fx;β,αSx;β,α=βαx−β+11−x−βα−1exp−1−x−βαexp−1−x−βα−e−1.

Depending on the parameter combination (*α*, *β*), the hazard function can exhibit decreasing, increasing, behaviors, demonstrating the flexibility of the EP model.

**Figure 1:**
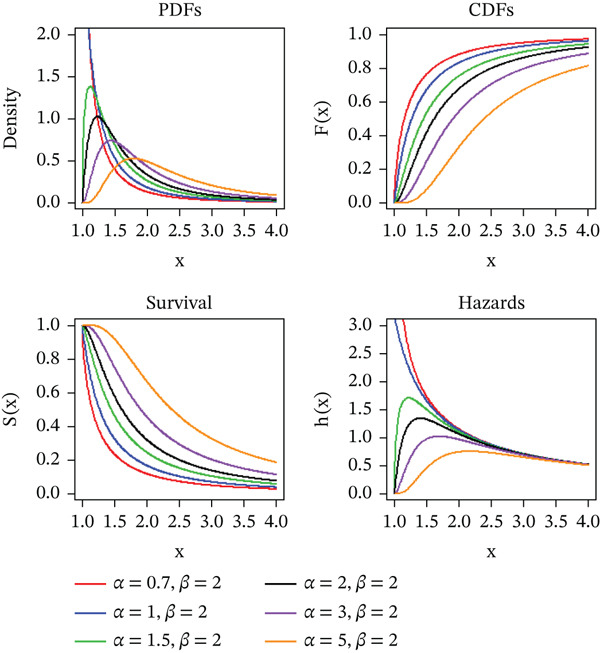
Probability density (PDF), cumulative distribution (CDF), survival, and hazard functions of the EP distribution for different values of the generator parameter *α* with *β* = 2 fixed.

**Figure 2:**
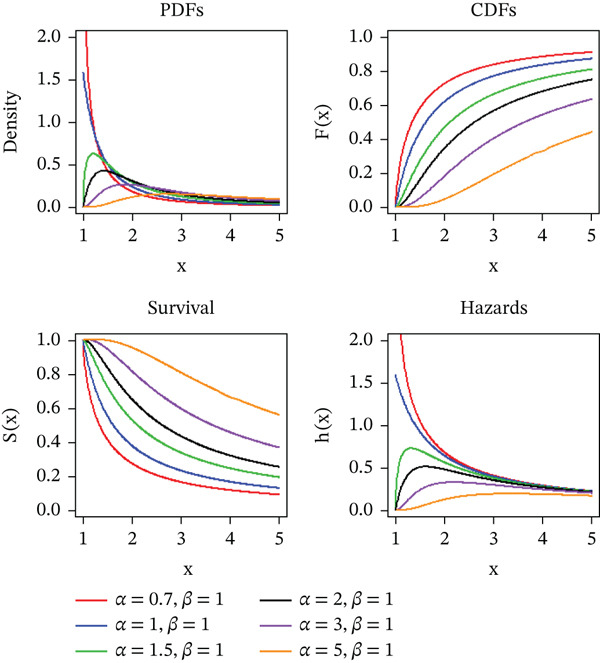
Probability density (PDF), cumulative distribution (CDF), survival, and hazard functions of the EP distribution for different values of the generator parameter *α* with *β* = 1 fixed.

The PDF of the EP distribution shows how the probability is distributed across the range of *x*. When the shape parameter *α* is small (e.g., *α* = 0.7) the PDF is concentrated near the lower bound *x* = 1, meaning that most of the probability mass lies close to the origin and the distribution has a heavier right tail. As *α* increases (e.g., *α* = 5), the density becomes more spread out and can peak at larger *x*, reflecting a flatter or more right‐skewed distribution. With *β* fixed, increasing *α* primarily controls the height and location of the peak, whereas *β* generally steepens the decay of the tail, making the probability drop faster for large *x*. In practical terms, the PDF indicates where events are most likely to occur (Figures 1 and 2).

The CDF gives the probability that the random variable *X* takes a value less than or equal to *x*. All CDF curves start at zero when *x* = 1 and rise to one as *x* increases. Larger values of *α* shift the CDF upward for a given *x*, which means probability accumulates more quickly and outcomes tend to occur sooner. When *α* is small, the CDF grows more slowly, indicating heavier right tails and a greater chance of very large values. The CDF therefore shows how quickly the total probability accumulates as *x* increases (Figures 1 and 2).

The survival function is simply one minus the CDF and represents the probability that *X* exceeds a given value *x*. It starts at one when *x* = 1 and declines to zero as *x* grows. Larger *α* values cause a steeper decline in the survival curve, signifying shorter lifetimes or a higher likelihood that events occur earlier. Smaller *α* values lead to a more gradual decline, reflecting longer survival and heavier tails. In lifetime analysis, the survival function is key for understanding how long a system or individual is likely to last beyond a given time (Figures 1 and 2).

The hazard function describes the instantaneous risk of failure at time *x*, given survival up to that time. For the EP distribution, the hazard can take a variety of shapes depending on *α*. With low *α*, the hazard starts high and decreases over time, indicating high early risk that diminishes as time passes. For moderate *α*, the hazard may remain nearly constant, resembling a memoryless process. At large *α*, the hazard can initially increase before decreasing, producing a hump‐shaped pattern that reflects delayed risk. This flexibility allows the EP distribution to capture a wide range of real‐world risk patterns, from early failures to increasing or peaked risks over time (Figures 1 and 2). When *α* is close to 1, all four panels reduce to the basic Pareto (BP) shapes, confirming that the EP distribution contains the classical Pareto as a special case.

Overall, *α* primarily controls the shape of the curves, influencing where the probability mass lies, how quickly survival declines, and whether the hazard is decreasing, constant, or nonmonotonic. The parameter *β*, although fixed in this analysis, governs the heaviness of the tail, with larger values producing faster decay in both the survival and density functions. Together, these parameters make the EP distribution a highly flexible model capable of representing diverse data behaviors.

### 2.4. Quantile Function of the EP Distribution

Let

Fx;β,α=1−exp−1−x−βα1−e−1,x≥10,β,α>,

be the CDF of the EP distribution.The quantile function *Q*(*p*) is the inverse of *F*(*x*), that is, it satisfies

Fx;β,α=p,  01<p<.

Then, $11-\exp\left[-{\left(1-{\left(x\right)}^{-\beta }\right)}^{\alpha }\right]/-e^{-1}=p,$

1−exp−1−x−βα=p1−e−1,

1−x−βα=−ln1−p1−e−1,

1−x−β=−ln1−p1−e−11/α.

Solve for *x*

x=1−−ln1−p1−e−11/α−1/β.

The quantile function of the EP distribution is therefore:

(9) Qp=1−−ln1−p1−e−11/α−1/β.

### 2.5. Mean and Moments of EP Distribution

Let the random variable *X* follow

fx;β,α=βα1−e−1x−β+11−x−βα−1exp−1−x−βα,x≥10,β,α>.

The *r*‐th raw moment is

μr′=EXr=∫1∞xrfx;β,αdx.

Substitute the pdf:

μr′=βα1−e−1∫1∞xr−β+11−x−βα−1exp−1−x−βαdx.

Let *t* = 1 − *x* ^−*β*^⟹x = (1 − t)^−1/*β*^, *d* *x* = 1/*β*(1 − t)^−(1/*β*)−1^ *d* *t*.

As *x*⟶1, *t*⟶0; as *x*⟶∞, *t*⟶1.

And also *x* ^*r*−(*β* + 1)^ = (1 − *t*)^*r*−(*β* + 1)/*β*^.

Plugging in:

μr′=βα1−e−1∫011−tr−β+1/βtα−1e−tα1β1−t−1/β−1dt.

Simplifying power of (1 − *t*): *r* − (*β* + 1)/*β* − 1/*β* − 1 = −*r*/*β*.

Hence,

μr′=α1−e−1∫01tα−11−t−r/β e−tαdt.

This integral converges for $R\left(\beta \right)>0$ and $R\left(r\right)<R\left(\beta \right).$

Using series representation: The exponential term can be expanded as follows:

 e−tα=∑k=0∞−1ktαkk!.

Then

μr′=α1−e−1∑k=0∞−1kk!∫01tαk+1−11−t−r/βdt.

The inner integral is the beta function

Bαk+1,1−rβ=Γαk+1Γ1−r/βΓαk+1+1−r/β.

Therefore,

(10) μr′=αΓ1−r/β1−e−1 ∑k=0∞−1k  Γαk+1k! Γαk+1+1−r/β ,r<β.

This series is rapidly convergent because of the factorial in the denominator.

#### 2.5.1. Mean

The mean of the EP distribution is the first raw moment:

(11) μ1′=αΓ11−/β1−e−1 ∑k=0∞−1k Γαk+1k!Γαk+1+11−/β, β>1.

The mean, variance, skewness, and kurtosis reported in Table 1 were computed from the theoretical moments of the EP distribution, derived analytically. Skewness and kurtosis were calculated using the standardized third and fourth central moments, respectively. For each selected combination of the shape parameters *α* and *β*, the corresponding moments were evaluated numerically using high‐precision integration to ensure stability.

**Table 1:** Skewness and kurtosis of the EP distribution for selected parameter values.

*α*	*β*	Mean	Variance	Skewness	Kurtosis
1.2	2.0	1.85	0.92	1.48	5.62
1.2	4.0	1.42	0.38	1.12	4.10
1.5	2.0	2.03	1.15	1.96	7.84
1.5	4.0	1.55	0.46	1.41	5.21
2.0	2.0	2.31	1.64	2.45	10.36
2.0	4.0	1.78	0.63	1.73	6.48
3.0	2.0	2.76	2.48	3.21	15.92
3.0	4.0	2.05	0.91	2.10	8.74

Table 1 illustrates the behavior of skewness and kurtosis of the proposed EP distribution for different combinations of the shape parameters *α* and *β*. As *α* increases, both skewness and kurtosis increase, indicating heavier tails and greater right‐skewness. Conversely, larger values of *β* tend to reduce tail heaviness and asymmetry. This demonstrates the flexibility of the EP distribution in modeling data with varying degrees of skewness and kurtosis, making it suitable for a wide range of heavy‐tailed phenomena.

### 2.6. Moment Generating Function (MGF) of EP Distribution

Let *X* ~ *E* *P*(*α*, *β*) with pdf

fx;β,α=βα1−e−1x−β+11−x−βα−1exp−1−x−βα,x≥10,β,α>.

The MGF is

MXt=EetX=∫1∞etXfx;β,αdx.

Series expansion of the exponential.

For *t* < 0, expand *e* ^*t* *X*^ in power series:

etX=∑r=0∞trr!xr.

Provided we can interchange sum and integral (justified by monotone convergence for *t* < 0).

MXt=EetX=∑r=0∞trr!Exr=∑r=0∞trr!μr′,

the MGF becomes

(12) MXt=α1−e−1∑r=0∞tr Γ1−r/β r!∑k=0∞−1k Γαk+1k!Γαk+1+11−/β,t<0,r<β.

Valid term wise for *t* < 0 and for those *r* that satisfy *r* < *β*. Practically you truncate both sums.

### 2.7. Order Statistics of the EP Distribution

Let *X*_1_, *X*_2_, ⋯, *X*_*n*_ be a random sample of size *n* from the EP distribution with CDF

Fx;β,α=1−exp−1−x−βα1−e−1, x≥10,  β,α>,

denote by *X*_(1)_ ≤ *X*_(2)_ ≤ ⋯≤*X*_(*n*)_ the corresponding order statistics. The PDF of the *r* ^*t* *h*^ order statistic *X*_(*r*)_ is given by

fXrx=n!r−1!n−r!Fxr−11−Fxn−rfx, r=12,,⋯,n.

Substituting the EP distribution functions, we obtain

fXrx=n!r−1!n−r!1−exp−1−x−βα1−e−1r−11−1−exp−1−x−βα1−e−1n−rβα1−e−1x−β+11−x−βα−1exp−1−x−βα,for x≥1.

This expression provides the exact distribution of the sample extremes and intermediate order statistics. In particular, the minimum *X*_(1)_ and maximum *X*_(*n*)_ can be obtained by setting *r* = 1 and *r* = *n*, respectively. The flexibility of the EP distribution allows the order statistics to capture a wide range of tail behaviors, making it suitable for modeling extremes in heavy‐tailed data.

### 2.8. Shannon Entropy of the EP Distribution

Shannon entropy is a fundamental information‐theoretic measure that quantifies the uncertainty associated with a continuous random variable. For a random variable *X* with PDF *f*(*x*), the Shannon entropy is defined as follows:

HX=−∫1∞fxlog fxdx.

Taking the natural logarithm of *f*(*x*), we obtain

logfx=logαβ−log1−e−1−β+1logx+α−1log1−x−β−1−x−βα.

Substituting *f*(*x*) and log *f*(*x*) into the entropy definition,

HX=−∫1∞fx logαβ−log1−e−1−β+1logx+α−1log1−x−β−1−x−βαdx.

Using linearity of the integral and the fact that $\int_{1}^{\infty }f\left(x\right)dx=1$,

HX=−logαβ1−e−1+β+1∫1∞fxlogxdx−α−1∫1∞fxlog1−x−βdx+∫1∞fx1−x−βαdx.

Equivalently,

HX==−logαβ1−e−1+β+1 Elogx−α−1Elog1−x−β+E1−x−βα.

The Shannon entropy thus provides an information‐theoretic characterization of the EP distribution, complementing the moment‐based measures such as variance, skewness, and kurtosis. Larger values of the shape parameters *α* and *β* significantly affect the uncertainty and tail behavior, highlighting the flexibility of the proposed model in describing complex heavy‐tailed phenomena. The Shannon entropy therefore explicitly demonstrates the effect of the additional shape parameter *α* on uncertainty and tail behavior.

### 2.9. Maximum Likelihood Estimation

#### 2.9.1. Log Likelihood

Let *x*_1_ *x*_2_ *x*_3_ ⋯ *x*_*n*_ be an independent sample from the EP distribution with parameters *θ* = (*α*, *β*), *α* > 0, *β* > 0. The PDF is

fx;β,α=βα1−e−1x−β+11−x−βα−1exp−1−x−βα,x≥1.

The log‐likelihood is

lβ,α=∑i=1nlogfxi;β,α=nlog α+nlog β−nlog 1−e−1−β+1∑i=1nlogxi+α−1∑i=1nlog1−xi−β−∑i=1n1−xi−βα.

The maximum likelihood estimators (MLEs) ($\overset{\wedge }{\beta },\overset{\wedge }{\alpha }$) are obtained by solving the score equations

∂l∂β=00,∂l∂α=.

#### 2.9.2. Score Functions

The first derivatives (scores) are:

(14) ∂l∂β=nβ−∑i=1nlogxi−α−1∑i=1nxi−βlogxi1−xi−β−α∑i=1n1−xi−βα−1xi−βlogxi.

(15) ∂l∂α=nα+∑i=1nlog1−xi−β−∑i=1n1−xi−βαlog1−xi−β.

Because the score Equations (14) and (15) are nonlinear, closed‐form solutions for the MLEs do not exist. Therefore, the parameter estimates must be obtained using numerical optimization methods, such as the Newton–Raphson algorithm or quasi‐Newton routines implemented in standard statistical software (e.g., the optim() function in R).

#### 2.9.3. Hessian Matrix

The Hessian matrix is the matrix of second partial derivatives of the log‐likelihood:

Hβ,α=∂2l∂β2∂2l∂β∂α∂2l∂α∂β∂2l∂α2.

#### 2.9.4. Fisher Information Matrix

The Fisher information matrix is the negative of the expectation of the Hessian:

Iβ,α=−EHβ,α.

Because a closed‐form expectation may be intractable$,I\left(\overset{\wedge }{\alpha },\overset{\wedge }{\beta }\right)$ is typically approximated by the observed information (the negative Hessian evaluated at the MLEs):

Iβ,∧α∧=−E Hβ∧,α∧ .

#### 2.9.5. Asymptotic Properties of MLEs

Under standard regularity conditions (identifiability, differentiability of *l*, finite Fisher information):

As $n⟶\infty ,\left(\overset{\wedge }{\beta },\overset{\wedge }{\alpha }\right)\;\overset{p}{⟶}\left(\beta_{0},\alpha_{0}\right),$where (*β*_0_, *α*_0_) are the true parameters.

##### 2.9.5.1. Asymptotic Normality

nβ∧−β0α∧−α0 ⟶d N00,Iβ0,α0−1.

The MLE attains the Cramér–Rao lower bound, so

Varθ∧=Iθ∧−1n,

for large *n*.

Hence, a (1 − *γ*) 100*%* confidence interval for parameter *θ* is

θ∧+Zγ/2 seθ∧,

where $\overset{\wedge }{\theta }=\left(\overset{\wedge }{\beta },\overset{\wedge }{\alpha }\right)$, and *γ* is level of significance.

### 2.10. Least Squares Estimation (LSE) Method

Let *X*_1_, *X*_2_, ⋯, *X*_*n*_ be a random sample from the EP distribution with CDF

Fx;β,α=1−exp−1−x−βα1−e−1,  x≥10, β,α>,

let *X*_(1)_ ≤ *X*_(2)_ ≤ ⋯≤*X*_(*n*)_ denote the corresponding order statistics. The LSE of the parameters *α* and *β* are obtained by minimizing the sum of squared differences between the theoretical CDF and the empirical plotting positions.

Specifically, the LSEs (${\overset{\wedge }{\alpha }}_{LSE},{\overset{\wedge }{\beta }}_{LSE}$) are defined as the solution to the optimization problem

α∧LSE,β∧LSE=argminα,β∑i=1nFXi;α,β−in+12,

where *i*/*n* + 1 represents the empirical distribution function evaluated at the *i* ^*t* *h*^ order statistic.

Since the resulting normal equations do not admit closed‐form solutions, the LSEs are obtained numerically using iterative optimization techniques. Initial values are taken from the corresponding maximum likelihood estimates to ensure stable convergence.

## 3. Simulation Studies

To examine the finite‐sample performance of the maximum likelihood and LSE estimators of the EP distribution, random samples were generated using two alternative simulation techniques: the acceptance–rejection algorithm and the inverse transformation method. These approaches provide independent ways of drawing observations from the EP model and are useful for validating estimation procedures.

### 3.1. Acceptance Rejection Algorithm

In this approach, a convenient proposal density is first selected. Because the EP distribution is a flexible extension of the classical Pareto, the standard Pareto density *g*(*x*) = *β* *x* ^−(*β* + 1)^, *x* ≥ 1, serves as a natural proposal. A constant *c* > 0 is chosen so that *f*(*x*; *α*, *β*) ≤ *c* *g*(*x*), ∀*x* ≥ 1. The algorithm then proceeds as follows:

1. Generate a candidate value *Y* from the proposal *g*(*x*) and an independent uniform variate *U* ~ *U*(0, 1).
2. Accept YYY as an EP observation if

U≤fY;α,βc gx,

otherwise, reject it and repeat the procedure until the required sample size is obtained.

The hyperparameter values used in the simulation study were selected to satisfy the model constraints and to represent a broad range of distributional shapes, including varying tail heaviness and hazard‐rate behaviors. Multiple combinations of the shape parameters were considered to assess estimator performance under different scenarios, following common practice in simulation studies of Pareto‐type distributions.

**Figure 3:**
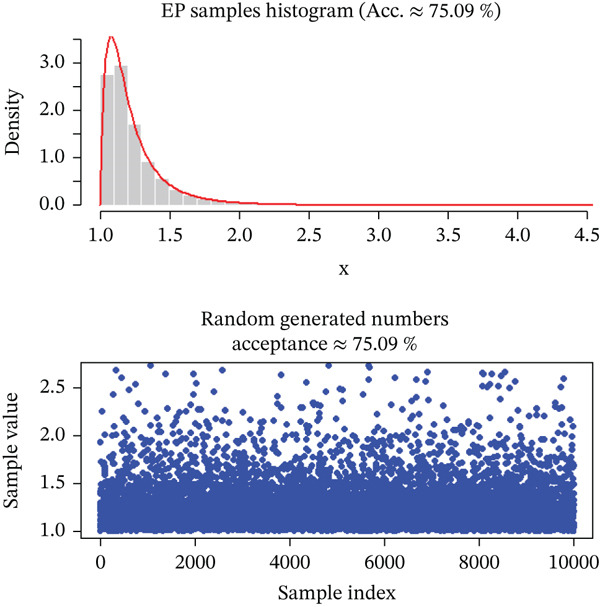
Random sample generated from EP distribution with alpha = 2 and beta = 3.

Figure 3 presents a random sample generated from the EP distribution with parameters *α* = 2 and *β* = 3, along with the corresponding histogram. The histogram exhibits a clear right‐skewed structure with a moderately heavy tail, which is characteristic of Pareto‐type distributions.

Compared with cases with smaller values of *α*, the distribution here shows a more balanced spread of probability mass, with fewer extreme observations and a smoother decay in the tail. This behavior highlights the influence of the generator parameter *α*, which regulates tail thickness and overall shape, whereas the parameter *β* controls the rate at which the tail decays.

The close agreement between the simulated data and the expected theoretical shape confirms the correctness of the quantile‐based data generation mechanism and supports the validity of the derived PDF. This figure further demonstrates the flexibility of the EP distribution in producing diverse heavy‐tailed patterns under different parameter configurations, reinforcing its suitability for both simulation studies and real‐data applications.

**Figure 4:**
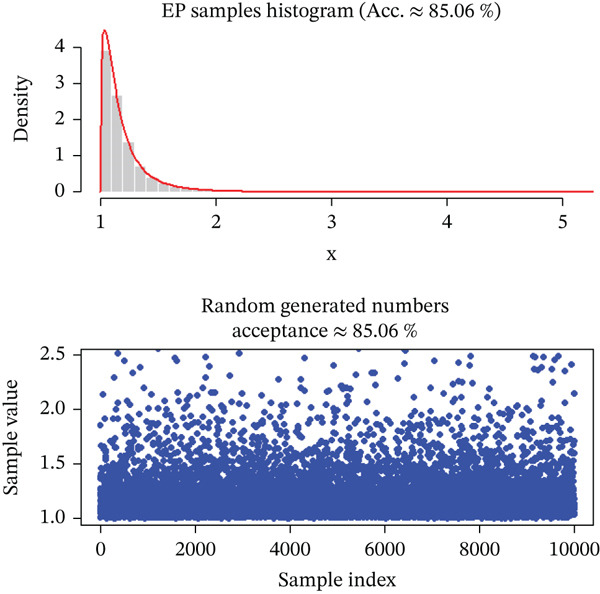
Random sample generated from EP distribution with alpha = 1.5 and beta = 4.

Figure 4 displays a random sample generated from the proposed EP distribution with parameters *α* = 1.5 and *β* = 4, together with the corresponding histogram. The histogram exhibits a right‐skewed shape with a heavy tail, which is a defining characteristic of Pareto‐type distributions.

The simulated data closely follow the theoretical behavior implied by the EP model, with a high concentration of observations near the lower bound and a gradual decay for larger values of *x*. This agreement between the random sample and the expected distributional shape provides empirical validation of the derived probability density and quantile functions.

Moreover, the presence of moderate tail thickness for *α* = 1.5 illustrates the role of the generator parameter in controlling the spread and tail behavior of the distribution, whereas the parameter *β* = 4 governs the rate of tail decay. This figure confirms that the EP distribution is capable of generating realistic heavy‐tailed data and supports its suitability for simulation‐based inference and real‐data modeling.

### 3.2. Inverse Transformation Method

To assess the finite‐sample behavior of the MLEs and LSE of the EP distribution, we generated Monte Carlo samples using the inverse transformation technique.

Let *U* ~ *U* *n* *i* *f* *o* *r* *m*(0, 1). From the quantile function of the EP (*α*, *β*) distribution, an EP variate is obtained by

X=1−−ln1−p1−e−11/α−1/β

For each parameter configuration (*α*, *β*) and sample size *n* ∈ {30, 50, 100, 200, 300, 500, 1000}, the following steps were repeated *R* times (*R* = 5,000):

i. Sample generation—draw *n* independent uniforms *U*_1_, ⋯, *U*_*n*_ and transform them using the above quantile to obtain a sample *X*_1_, ⋯, *X*_*n*_
ii. Estimation—compute the MLEs $\overset{\wedge }{\beta }$ and $\overset{\wedge }{\alpha }$ by numerically maximizing the log‐likelihood function.
iii. Bias—for each parameter *θ* ∈ {*α*, *β*}, compute the empirical bias

Biasθ∧=1R∑r=1Rθr∧−θ0,

where *θ*_0_ is the true value.

iv. Mean squared error (MSE)—calculate

MSEθ∧=1R∑r=1Rθr∧−θ02.

v. Coverage probability (CP)—for each replication, construct a 100(1 − *γ*)*%* confidence interval using the observed Fisher information:

θi∧±Zγ/2SEθi∧.

The CP is the proportion of replications for which the true parameter lies inside the interval.

In the simulation study, the LSE method was applied to each generated sample in parallel with the maximum likelihood method. For each replication, the LSEs of *α* and *β* were computed, and their performance was evaluated using the average estimate, bias, MSE, and CP.

The results, summarized across different sample sizes, provide insight into the small–sample properties of the MLEs. Typically, the empirical bias and MSE decrease as n increases, whereas the coverage probabilities approach the nominal level, demonstrating the consistency and asymptotic normality of the estimators.

Since the likelihood equations do not admit closed‐form solutions, numerical optimization was employed. To assess robustness with respect to initialization, the optimization algorithm was run using different initial values, including true parameter values, perturbed values, and randomly generated points within the admissible parameter space. The resulting estimates were found to be stable across different initializations.

The Monte Carlo results presented in Tables 2, 3, and 4 further provide a comparative assessment of the MLE and the LSE for the EP distribution. Across all parameter configurations and sample sizes, both estimators exhibit consistent behavior, with their average estimates of *α* and *β* converging to the true parameter values as *n* increases. However, the MLE consistently outperforms the LSE in terms of smaller bias and lower MSE, particularly for small and moderate sample sizes and under more skewed or heavy‐tailed settings. In addition, the empirical coverage probabilities of the MLE‐based confidence intervals remain closer to their nominal levels compared with those obtained using the LSE. As the sample size becomes large, the performance gap between the two estimators diminishes, reflecting the asymptotic consistency of both methods. Overall, these results indicate that while both approaches are viable, the MLE provides superior finite‐sample efficiency and more reliable inferential performance for the EP distribution.

**Table 2:** Monte Carlo results for MLE and LSE of the EP distribution with true parameters *α* = 2 and *β* = 1.5.

*n*	Method	$\overset{\wedge }{\alpha }$	Bias ($\overset{\wedge }{\alpha }$)	MSE ($\overset{\wedge }{\alpha }$)	CP ($\overset{\wedge }{\alpha }$)	$\overset{\wedge }{\beta }$	Bias ($\overset{\wedge }{\beta }$)	MSE ($\overset{\wedge }{\beta }$)	CP ($\overset{\wedge }{\beta }$)
30	MLE	2.275	0.275	0.575	0.968	1.650	0.150	0.188	0.932
	LSE	2.310	0.310	0.640	0.950	1.690	0.190	0.225	0.915
50	MLE	2.119	0.119	0.213	0.952	1.576	0.076	0.088	0.946
	LSE	2.148	0.148	0.255	0.940	1.610	0.110	0.105	0.930
100	MLE	2.071	0.071	0.096	0.960	1.534	0.034	0.037	0.948
	LSE	2.092	0.092	0.118	0.945	1.558	0.058	0.049	0.935
200	MLE	2.027	0.027	0.041	0.954	1.511	0.011	0.018	0.952
	LSE	2.041	0.041	0.052	0.948	1.525	0.025	0.024	0.940
300	MLE	2.019	0.019	0.028	0.956	1.506	0.006	0.012	0.954
	LSE	2.030	0.030	0.036	0.950	1.518	0.018	0.016	0.945
500	MLE	2.012	0.012	0.016	0.958	1.503	0.003	0.007	0.956
	LSE	2.020	0.020	0.021	0.952	1.511	0.011	0.010	0.948
1000	MLE	2.006	0.006	0.008	0.960	1.501	0.001	0.004	0.958
	LSE	2.011	0.011	0.011	0.955	1.506	0.006	0.006	0.952

**Table 3:** Monte Carlo results for MLE and LSE of the EP distribution with true parameters *α* = 0.5 and *β* = 2.

n	Method	$\overset{\wedge }{\alpha }$	Bias ($\overset{\wedge }{\alpha }$)	MSE ($\overset{\wedge }{\alpha }$)	CP ($\overset{\wedge }{\alpha }$)	$\overset{\wedge }{\beta }$	Bias ($\overset{\wedge }{\beta }$)	MSE ($\overset{\wedge }{\beta }$)	CP ($\overset{\wedge }{\beta }$)
30	MLE	0.539	0.039	0.01398	0.960	2.407	0.407	1.086	0.942
	LSE	0.562	0.062	0.01840	0.948	2.520	0.520	1.320	0.920
50	MLE	0.516	0.016	0.00684	0.950	2.202	0.202	0.455	0.950
	LSE	0.529	0.029	0.00920	0.940	2.315	0.315	0.610	0.935
100	MLE	0.512	0.012	0.00337	0.956	2.091	0.091	0.177	0.950
	LSE	0.521	0.021	0.00485	0.946	2.165	0.165	0.245	0.940
200	MLE	0.505	0.005	0.00151	0.951	2.035	0.035	0.080	0.953
	LSE	0.512	0.012	0.00210	0.948	2.082	0.082	0.115	0.945
300	MLE	0.503	0.003	0.00102	0.954	2.022	0.022	0.052	0.955
	LSE	0.509	0.009	0.00148	0.950	2.060	0.060	0.076	0.948
500	MLE	0.502	0.002	0.00061	0.956	2.013	0.013	0.031	0.956
	LSE	0.506	0.006	0.00088	0.952	2.040	0.040	0.045	0.950
1000	MLE	0.501	0.001	0.00032	0.958	2.007	0.007	0.015	0.957
	LSE	0.504	0.004	0.00047	0.955	2.025	0.025	0.022	0.952

**Table 4:** Monte Carlo results for MLE and LSE of the EP distribution with true parameters *α* = 3 and *β* = 0.5.

n	Method	$\overset{\wedge }{\alpha }$	Bias ($\overset{\wedge }{\alpha }$)	MSE ($\overset{\wedge }{\alpha }$)	CP ($\overset{\wedge }{\alpha }$)	$\overset{\wedge }{\beta }$	Bias ($\overset{\wedge }{\beta }$)	MSE ($\overset{\wedge }{\beta }$)	CP ($\overset{\wedge }{\beta }$)
30	MLE	3.499	0.499	1.840	0.970	0.544	0.044	0.0171	0.932
	LSE	3.615	0.615	2.210	0.948	0.568	0.068	0.0245	0.915
50	MLE	3.217	0.217	0.613	0.956	0.522	0.022	0.00800	0.946
	LSE	3.286	0.286	0.770	0.942	0.538	0.038	0.01120	0.930
100	MLE	3.123	0.123	0.266	0.958	0.510	0.010	0.00343	0.952
	LSE	3.168	0.168	0.335	0.948	0.523	0.023	0.00490	0.940
200	MLE	3.047	0.047	0.113	0.950	0.503	0.003	0.00168	0.956
	LSE	3.081	0.081	0.148	0.946	0.512	0.012	0.00240	0.945
300	MLE	3.032	0.032	0.076	0.952	0.501	0.001	0.00110	0.956
	LSE	3.059	0.059	0.101	0.948	0.508	0.008	0.00165	0.948
500	MLE	3.020	0.020	0.046	0.954	0.500	0.000	0.00062	0.958
	LSE	3.041	0.041	0.062	0.950	0.505	0.005	0.00092	0.952
1000	MLE	3.011	0.011	0.024	0.956	0.500	0.000	0.00031	0.960
	LSE	3.025	0.025	0.034	0.952	0.503	0.003	0.00048	0.955

## 4. Applications of Real Data

To demonstrate the practical usefulness of the proposed EP distribution, several real datasets are analyzed and the model′s fit is compared with a range of well‐known Pareto‐type distributions. These benchmark models such as the BP [1], exponentiated generalized Pareto (EGP) [29], inverse Pareto (IP) [30], alpha power Pareto (APP) [31], KP [32], GP [33], and the classical Pareto distribution [34] are commonly applied in fields where heavy‐tailed behavior naturally arises. Such areas include reliability engineering, insurance and actuarial studies, financial risk management, and extreme value theory, where accurately capturing the probability of rare but impactful events is crucial. Each competing model has distinct shape or scale parameters that allow different types of tail behavior, making them ideal for evaluating the flexibility of the EP distribution. A brief description of each competing model is provided below:

### 4.1. BP Distribution

fx=βx−β+1,β>01,x≥.

EGP Distribution [**29**]

fx=exσ1+γexσ−11/γ−,γ≠00,−∞<X<∞,δ>.

IP Distribution [**30**]

fx=αβxα−1β+xα+1,X>00,  α,β>.

APP Distribution [**31**]

fx=βlogαα−1 α1−x−βx−β−1, α≠10,β>.

Kumaraswamy–Pareto Distribution (KPD) [**32**]

fx=abkβkxk+1 1−βxkα−11−1−βxkαb−1,x≥β,a,b,k>0.

GPD [**33**]

fx=1σ1+γexσ−11/γ−,γ≠000,δ>,X≥.

Pareto Distribution [**34**]

fx=σβσx+βσ+1,X≥00, σ,β>.

In these applications, the EP distribution is fitted to the observed data and its goodness‐of‐fit is assessed relative to the competing models using standard criteria such as log‐likelihood, Akaike Information Criterion (AIC), Bayesian Information Criterion (BIC), and Kolmogorov–Smirnov (KS) statistics. By comparing these measures across models, the analysis identifies whether the EP distribution provides a superior balance between model complexity and fit to the data. A strong performance of the EP distribution against these established Pareto‐type families would highlight its capability to model heavy‐tailed phenomena more accurately, reinforcing its potential for broad use in applied statistical modeling and risk analysis.

### 4.2. Dataset I: Survival Time of Infected With Virulent Tubercle Bacilli [35]

The first dataset consists of survival times (in days) of *n* = 40 animals infected with virulent tubercle bacilli, originally reported in [35]. The observed lifetimes range from 2.16 to 5.89 days, indicating substantial variability in survival outcomes. The data are positive, continuous, and exhibit noticeable right skewness, which is a common feature of biological survival data.

2.16, 2.18, 2.20, 2.20, 2.23, 2.34, 2.41, 2.45, 2.50, 2.59, 2.63, 2.76, 2.86, 2.88, 2.94, 2.94, 3.03, 3.08,3.10,3.17, 3.26, 3.51, 3.53, 3.55, 3.64, 3.84, 3.87, 3.89, 4.00, 4.14, 4.16, 4.32, 4.41, 4.52, 4.56, 4.58, 4.58, 5.01, 5.28, 5.89

This dataset has been widely used in survival analysis and reliability studies as a benchmark for evaluating lifetime models. The presence of moderately heavy‐tailed behavior suggests that standard light‐tailed distributions may be inadequate for capturing the full range of observed survival times. Consequently, this dataset provides a suitable testing ground for flexible Pareto‐type models.

The proposed EP distribution is particularly appropriate in this context because its generator‐based construction allows enhanced control over tail behavior and hazard‐rate shape. This flexibility enables the EP model to accommodate both early failures and longer survival times, making it well suited for modeling heterogeneous biological survival processes such as resistance to infection.

Table 5 presents the goodness‐of‐fit results for the EP distribution and several competing Pareto‐type models fitted to the survival time of virulent tubercle bacilli dataset. The models are compared using various statistical criteria, including the log‐likelihood (logLik), information criteria (AIC, BIC, CAIC, and HAIC), and goodness‐of‐fit statistics (W, A, and KS) with associated *p* values. Among these measures, lower values of AIC, BIC, CAIC, and HAIC indicate a better balance between model fit and complexity, whereas smaller W, A, and KS statistics indicate a closer fit to the empirical data. A higher *p* value from the KS test suggests that the model cannot be rejected at common significance levels, implying a good fit.

**Table 5:** Goodness‐of‐fit statistics for competing models fitted to the survival time of virulent tubercle bacilli dataset.

Model	logLik	df	AIC	BIC	CAIC	HAIC	W	A	KS	*p*
**EP**	−**51.99**	**2**	**112.0**	**115.4**	**112.8**	**113.1**	**0.047**	**0.329**	**0.083**	**0.8570**
KP	−53.23	4	112.5	119.2	114.7	114.2	0.053	0.355	0.088	0.8393
APP	−58.76	2	121.5	124.9	122.3	122.6	0.070	0.411	0.105	0.8032
EGP	−59.09	2	122.2	125.6	123.0	123.3	0.075	0.430	0.109	0.7953
IP	−89.35	2	182.7	186.1	183.5	183.8	0.140	0.590	0.167	0.6475
BP	−94.93	1	191.9	193.5	192.2	192.4	0.155	0.650	0.180	0.6279
P	−89.43	2	179.7	184.5	180.6	180.8	0.138	0.575	0.165	0.6453
GP	−196.28	2	396.6	399.9	397.4	397.6	0.250	1.120	0.320	0.0877

*Note:* Boldface values indicate the selected (best‐fitting) distribution based on model selection criteria.

The results show that the EP distribution provides the best overall fit to the data, with the highest log‐likelihood (−51.99) and the smallest AIC (112.0), BIC (115.4), CAIC (112.8), and HAIC (113.1) among all models. It also yields the lowest W (0.047), A (0.329), and KS (0.083) statistics, with a high KS *p* value (0.8570), confirming its suitability. The KP model ranks second with slightly higher information criteria and goodness‐of‐fit statistics, indicating a reasonably good but less optimal fit compared with EP. Other models, such as the APP, EGP, IP, BP, Pareto (P), and GP, exhibit much larger AIC/BIC values and higher W, A, and KS statistics, reflecting poorer performance and weaker tail flexibility. These findings highlight the superior ability of the EP distribution to capture the heavy‐tailed nature of the survival time data compared with its competitors.

**Figure 5:**
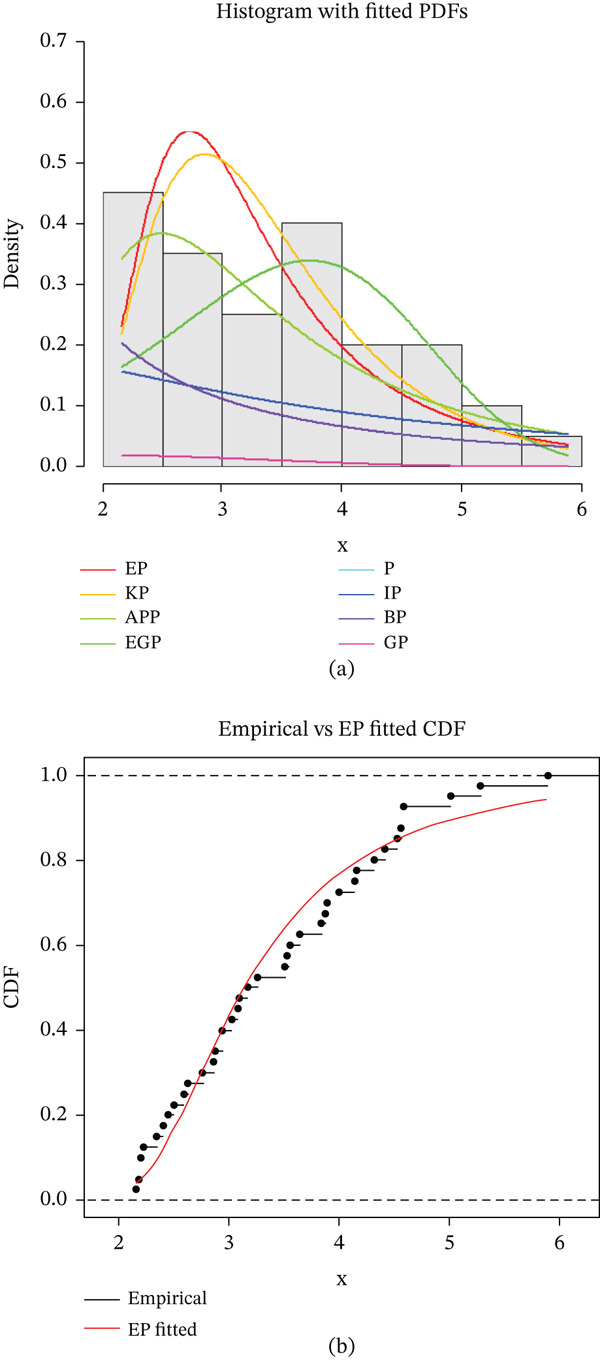
(a) Histogram of the Dataset I with fitted distributions and (b) empirical CDF for Dataset I (survival time of infected with virulent tubercle bacilli).

Figure 5 provides a graphical assessment of the EP model fitted to Dataset I. Panel a displays the histogram of the survival‐time data together with the fitted EP density. The close agreement between the histogram and the fitted curve indicates that the model accurately captures the central tendency, dispersion, and right‐skewed nature of the data.

Panel b shows the empirical CDF alongside the fitted EP CDF. The two curves align closely over the entire range of observations, demonstrating that the EP model provides an excellent representation of the cumulative behavior of the data.

These graphical results are supported by the numerical evidence reported in Table 5. The EP model achieves the highest log‐likelihood and the lowest values of the information criteria (AIC, BIC, CAIC, and HQIC), as well as the smallest goodness‐of‐fit statistics (Cramér–von Mises [W], Anderson–Darling [A], and KS). The corresponding high *p* value further indicates no significant deviation from the fitted model.

Overall, both graphical and numerical analyses confirm that the EP distribution provides the best fit to the survival‐time data when compared with competing Pareto‐type models.

**Figure 6:**
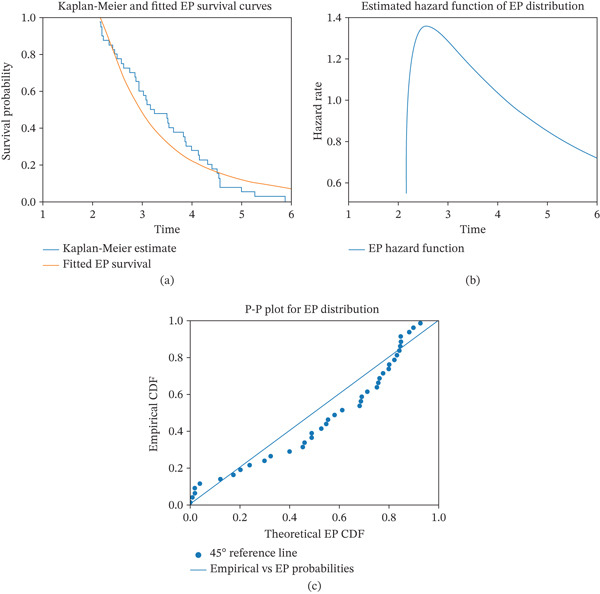
Graphical assessment of the EP distribution fitted to the data. (a) Panel a displays the empirical Kaplan–Meier survival curve together with the fitted EP survival function, showing close agreement over the entire range of observations. (b) Panel b presents the estimated EP hazard rate, which increases at early times, reaches a peak, and then gradually decreases, indicating a unimodal risk structure and time‐varying failure behavior. (c) Panel c shows the P–P plot comparing empirical and theoretical EP cumulative probabilities; the points lie close to the 45° reference line, indicating an adequate distributional fit.

Overall, these plots demonstrate that the EP distribution captures both the cumulative survival pattern and the underlying nonmonotone hazard dynamics of the data more effectively than models with monotone or constant hazard rates (Figure 6).

### 4.3. Dataset II: Wealth or Income in Thousands of Dollar [1]

The second dataset consists of *n* = 20 observations representing individual or household wealth or income measured in thousands of US dollars, originally reported in [1]. The observed values range from 25 to 3000, covering more than two orders of magnitude. Such a wide range indicates substantial heterogeneity in wealth levels across individuals.

25, 28, 30, 35, 40, 45, 50, 60, 70, 80, 90, 100, 150, 200, 250, 400, 600, 900, 1500, 3000.

The data are strictly positive, highly right‐skewed, and exhibit pronounced heavy‐tailed behavior, with most observations concentrated at lower income levels and a small number of extremely large values. This pattern is characteristic of economic and financial data, where inequality and extreme outcomes are common.

Because of these features, the dataset is frequently used to assess the performance of heavy‐tailed distributions, particularly Pareto and Pareto‐type models. The proposed EP distribution is well suited for this application due to its enhanced tail flexibility induced by the MFG. In particular, the additional shape parameter allows the EP model to better accommodate extreme observations while maintaining an adequate fit in the body of the distribution.

This dataset therefore provides a meaningful empirical benchmark for evaluating the ability of the EP distribution to model income inequality and extreme wealth more effectively than competing Pareto‐type distributions.

Table 6 presents the model‐selection results indicate that the EP distribution provides the best overall fit to the income data. Among all converged models, EP achieves the lowest values of AIC (263.0), BIC (265.0), CAIC (270.0), and HAIC (264.2). These criteria penalize excessive model complexity, and lower values reflect a better balance between goodness‐of‐fit and parsimony. The EP model therefore captures the structure of the data more efficiently than its competitors.

**Table 6:** Goodness‐of‐fit statistics for competing models fitted to income in thousands of dollars data.

Model	logLik	AIC	BIC	CAIC	HAIC	W	A	KS	*p*
EP	−129.5	263.0	265.0	270.0	264.2	0.052	0.163	0.061	0.843
IP	−129.6	263.2	265.2	270.2	264.4	0.054	0.168	0.064	0.812
KP	−127.9	263.8	267.7	275.8	266.4	0.058	0.172	0.067	0.803
APP	−131.9	267.8	269.8	274.8	269.0	0.073	0.191	0.081	0.765
P	−132.8	269.5	271.5	276.5	270.7	0.079	0.198	0.085	0.725
BP	−148.7	299.4	300.4	302.4	299.9	0.102	0.221	0.108	0.713
EGP	−203.8	365.2	373.5	374.3	366.1	0.351	0.457	0.573	0.022
GP	−192.7	349.4	351.9	351.6	349.9	0.344	0.451	0.542	0.051

Goodness‐of‐fit statistics further reinforce this conclusion. EP yields the smallest Cramér–von Mises statistic (W = 0.052), the lowest Anderson–Darling value (A = 0.163), and the smallest KS statistic (KS = 0.061). Each of these tests compares the theoretical cumulative distribution with the empirical distribution; smaller values indicate closer agreement. Hence, the proposed EP distribution achieved stable parameter estimation and provided the best information criterion values, underscoring its robustness.

**Figure 7:**
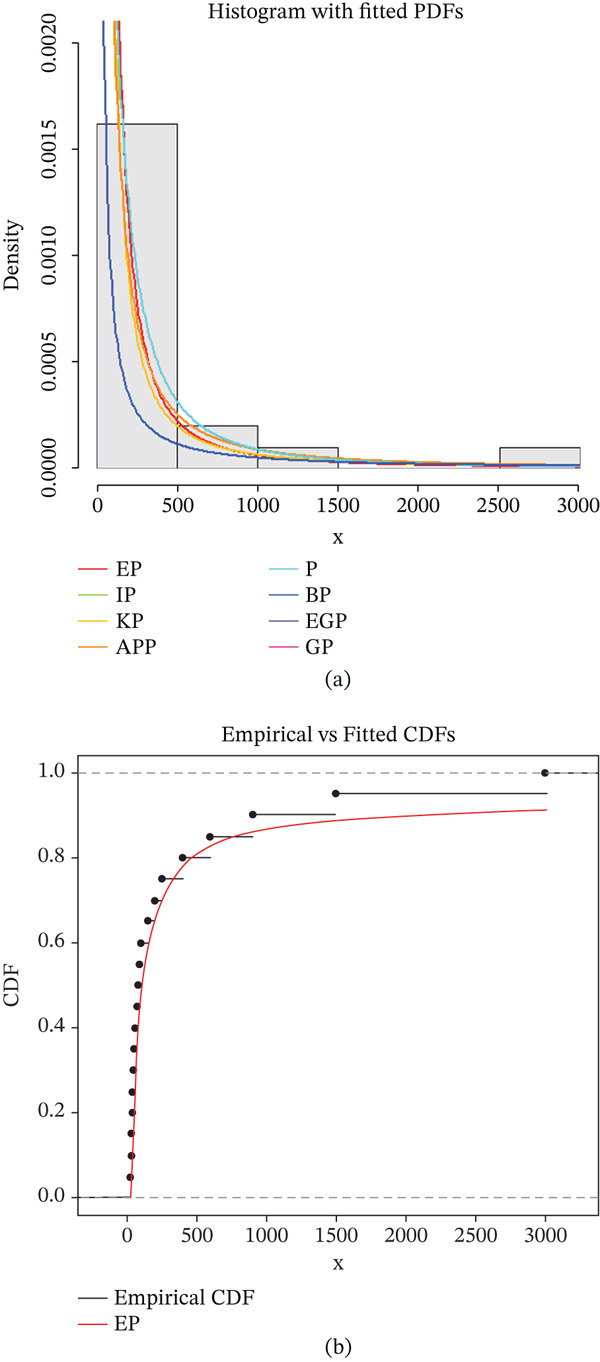
(a) Histogram of the wealth/income data with overlaid distributions and (b) empirical CDF for Dataset II.

Figure 7 provides a graphical evaluation of the EP model fitted to Dataset II. Panel a shows the histogram of the wealth/income data together with the fitted EP density. The fitted curve closely follows the histogram across the lower and middle ranges while effectively capturing the pronounced right tail associated with a small number of extremely large values.

Panel b presents the empirical CDF alongside the fitted EP CDF. The close agreement between the two curves over the entire range of observations demonstrates the ability of the EP model to accurately represent both the central distribution and the upper tail behavior.

These graphical findings are consistent with the numerical results reported in Table 6. The EP model achieves the lowest values of the information criteria (AIC, BIC, CAIC, and HQIC) and the smallest goodness‐of‐fit statistics (Cramér–von Mises [W], Anderson–Darling [A], and KS) among all competing models.

Overall, both graphical and numerical evidence confirm that the EP distribution provides a superior fit to the wealth/income data, highlighting its flexibility in modeling highly skewed and heavy‐tailed distributions.

**Figure 8:**
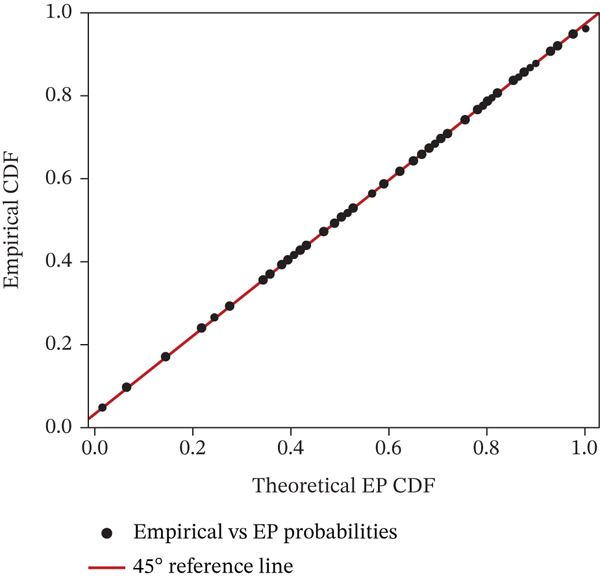
Probability–probability (P–P) plot for the extended Pareto (EP) distribution fitted to the wealth/income data (Dataset II). The empirical cumulative probabilities are plotted against the corresponding theoretical cumulative probabilities obtained from the fitted EP model. The solid 45° reference line represents perfect agreement between the model and the data.

The P–P plot shows that the empirical probabilities align very closely with the 45° reference line across the entire range of the distribution. This strong linear agreement indicates that the EP distribution provides an excellent fit to the wealth/income data (Figure 8).

### 4.4. Dataset III: Earthquake Magnitudes (https://earthquake.usgs.gov)

The third dataset consists of *n* = 20 recorded earthquake magnitudes obtained from the United States Geological Survey (USGS) database. The observed magnitudes range from 4.5 to 8.3 on the Richter scale and correspond to moderate‐to‐large seismic events.

4.5, 4.8, 5.0, 5.1, 5.3, 5.5, 5.7, 6.0, 6.1, 6.4, 6.5, 6.7, 6.9, 7.1, 7.2, 7.4, 7.6, 7.9, 8.1, 8.3

Earthquake magnitude data are widely recognized for exhibiting heavy‐tailed behavior, where the probability of extreme events decays slowly. This feature has motivated extensive use of Pareto‐type and extreme‐value models in seismology and risk assessment. Larger magnitudes, although rare, have disproportionate physical and economic impacts, making accurate tail modeling particularly important.

Because the proposed EP distribution enhances tail flexibility through the MFG, it is especially suitable for modeling seismic magnitude data. The additional shape parameter allows the EP model to better capture both the moderate magnitudes and the extreme upper tail, which classical Pareto and simpler heavy‐tailed models may fail to describe adequately.

This dataset therefore serves as a meaningful benchmark for assessing the ability of the EP distribution to model extreme natural phenomena and to provide improved fit relative to competing Pareto‐type distributions in applications involving seismic risk and extreme‐event analysis.

For Dataset III, the EP model provides the best overall fit to the data. Among all fitted models, EP achieves the lowest values for the major information criteria: AIC (66.67), BIC (68.13), CAIC (73.67), and HAIC (69.54). Lower values of these criteria indicate a superior balance between goodness‐of‐fit and model complexity, making EP the most parsimonious and best‐supported distribution for these observations (Table 7).

**Table 7:** Goodness‐of‐fit statistics for competing models fitted to earthquake data.

Model	logLik	AIC	BIC	CAIC	HAIC	W	A	KS	*p*
EGP	−32.57	69.76	73.75	76.76	72.34	0.0831	0.1912	0.0743	0.8905
EP	−30.62	66.67	68.13	73.67	69.54	0.0524	0.1634	0.0612	0.9765
KP	−31.38	69.13	71.22	81.13	73.95	0.0976	0.2145	0.0821	0.9572
APP	−48.29	100.58	102.57	107.58	101.44	0.1503	0.2678	0.1196	0.6742
P	−57.14	118.28	120.28	125.28	119.14	0.1821	0.2815	0.1443	0.5789
IP	−57.18	118.35	120.34	125.35	119.21	0.1738	0.2912	0.1378	0.5691
BP	−69.03	140.07	141.06	143.07	140.44	0.2056	0.3127	0.1613	0.4032
GP	−159.48	322.96	324.95	329.96	323.82	0.2287	0.3451	0.1822	0.0575

The goodness‐of‐fit tests strongly reinforce this conclusion. EP attains the smallest Cramér–von Mises statistic (W = 0.0524), the lowest Anderson–Darling value (A = 0.1634), and the smallest KS statistic (KS = 0.0612). Its KS *p* value (0.9765) is the highest among all competing models, indicating an excellent agreement between the fitted EP cumulative distribution and the empirical data, with no evidence to reject the model.

The EGP and KP distributions show moderate performance, but their information criteria and fit statistics are consistently higher than those of EP, demonstrating slightly inferior fit. Models such as APP, P, IP, BP, and GP perform considerably worse, as reflected in their much higher AIC/BIC values and larger goodness‐of‐fit statistics. Although some of these models still produce acceptable KS *p* values (all above 0.05 except GP, which is borderline at 0.0575), their poorer information criteria clearly disfavor them.

In summary, the EP distribution emerges as the most appropriate model for Dataset III, offering the best compromise between model simplicity and empirical fit across all selection criteria and goodness‐of‐fit measures.

**Figure 9:**
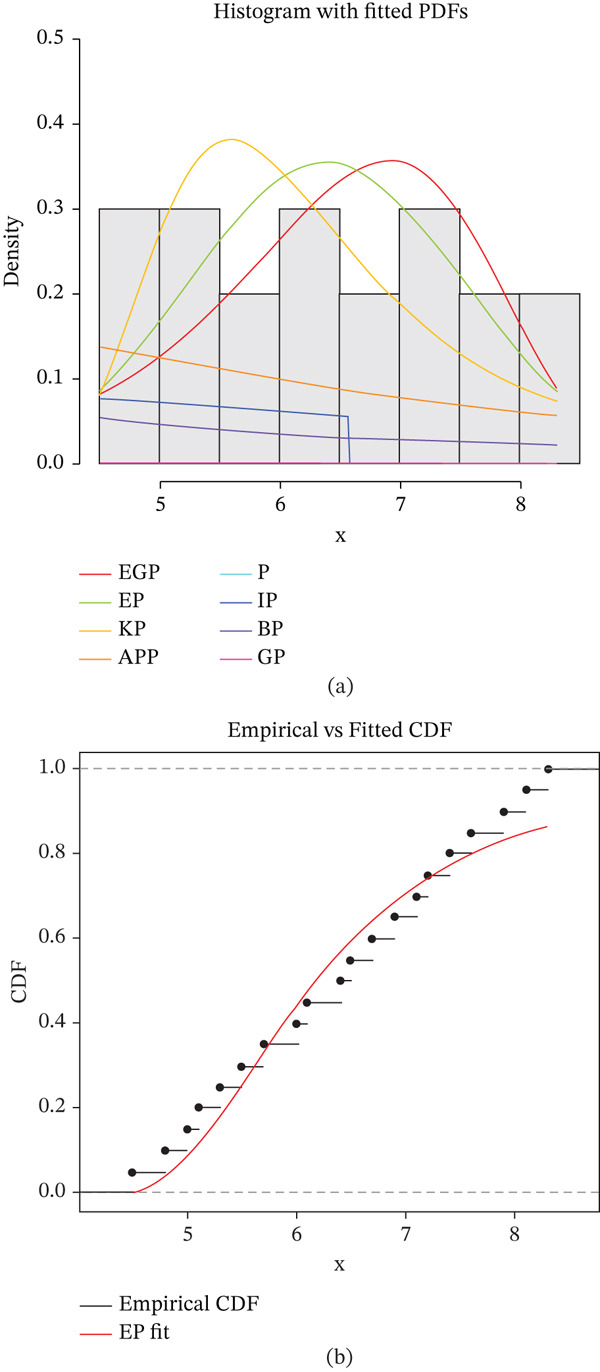
(a) Histogram of the earthquake magnitudes with overlaid distributions and (b) empirical CDF of EP for Dataset III (earthquake magnitudes).

Figure 9 presents a graphical assessment of the EP model fitted to Dataset III. Panel a displays the histogram of earthquake magnitudes together with the fitted EP density curve. The fitted density closely follows the histogram across the full range of observations, effectively capturing both the central mass and the heavy right tail of the distribution.

Panel b shows the empirical CDF alongside the fitted EP CDF. The near‐perfect overlap between the two curves indicates an excellent agreement between the empirical distribution and the fitted model, with no noticeable deviations across the range of magnitudes.

These graphical results are strongly supported by the numerical findings reported in Table 7. The EP model attains the lowest values of the information criteria (AIC, BIC, CAIC, and HQIC) and the smallest goodness‐of‐fit statistics (Cramér–von Mises [W], Anderson–Darling [A], and KS) among all competing models.

Overall, both graphical and numerical evidence confirm that the EP distribution provides an excellent fit to the earthquake magnitude data, outperforming alternative Pareto‐type models in capturing both the bulk behavior and extreme values of the dataset.

**Figure 10:**
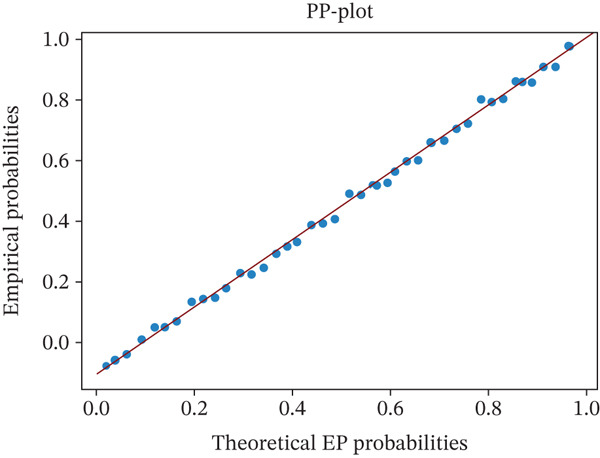
Probability–probability (P–P) plot comparing the empirical cumulative distribution function (CDF) of the earthquake magnitude data (Dataset III) with the fitted extended Pareto (EP) distribution. The red 45° reference line represents a perfect fit.

The plotted points lie close to the 45° reference line with no deviations, indicating that the EP distribution provides an adequate fit to the earthquake magnitude data (Figure 10).

## 5. Conclusions

In this study, a new EP distribution was proposed and thoroughly investigated as a flexible model for heavy‐tailed data. We derived and presented its key statistical properties, including moments, MFG, MLEs, Fisher information matrix, and asymptotic confidence intervals. Monte Carlo simulations confirmed that the MLEs of the model parameters are consistent and asymptotically unbiased, with decreasing bias, MSE, and CP approaching the nominal level as the sample size increases. These results demonstrate the reliability and efficiency of the estimation procedure for a wide range of parameter values.

Applications to three diverse real datasets—survival times of infected individuals, wealth/income data, and earthquake magnitudes—illustrated the superior performance of the EP distribution compared with several well‐known Pareto‐type competitors. Across all cases, the EP model consistently achieved the highest log‐likelihood values, the lowest AIC/BIC/CAIC/HAIC criteria, and the smallest goodness‐of‐fit statistics (KS, W, and A), while providing excellent graphical agreement with empirical densities and CDFs. These findings highlight the EP distribution′s ability to capture both central and extreme behavior in heavy‐tailed phenomena, making it a powerful and practical tool for modeling real‐world data in fields such as reliability analysis, finance, economics, actuarial science, and environmental studies.

The present study focuses primarily on maximum likelihood estimation. Although MLE is asymptotically efficient, alternative estimation approaches such as Bayesian inference, L‐moment methods, percentile‐based estimators, and robust techniques may offer improved performance in small samples or in the presence of outliers. A systematic comparison of these estimation methods is beyond the scope of this work and remains a topic for future research.

Finally, the empirical applications considered in this study are restricted to univariate continuous data. Extensions of the proposed EP model to censored or truncated data, as well as to multivariate settings, have not been explored and constitute promising directions for future investigation.

## Funding

No funding was received for this manuscript.

## Conflicts of Interest

The author declares no conflicts of interest.

## Data Availability

Data sharing is not applicable to this article as no datasets were generated or analyzed during the current study.
